# Letter from Accoucheur, No. 2

**Published:** 1845-11-01

**Authors:** 


### Letter from Accoucheur, No. 2.

Communicated for the Buffalo Medical Journal.

Educated obstetricians of this time know little of what was the practice in that department forty years ago, or what it is now in many parts of the country. To be known it must have been seen, for one will seek in vain for any historical records. Forty years ago, our knowledge of anatomy was very imperfect, of physiology even more so. We had no schools, and but few books; and many a man assumed the responsibilities of a Physician, and entered on the practice of Physic, Surgery and Midwifery, with no other knowledge of either, than what he had derived from the oral communications of some older country physician. The modicum of reward, too, for professional services was altogether inadequate to provide the means of subsistence; and the Doctor, of necessity, was a practical farmer, a mechanic, or a tradesman of some sort, to enable him to live. No wonder, then, that the practice of physic was mostly empirial.

The first object of a country physician when he "set up," was to secure the obstetrical practice "in his ride," as this was the key to all other medical employment and preferment; and if he was shrewd enough to ingratiate himself with a few, clever, female gossips, the "way was his own." The practice was desirable, too, on other accounts. The fees, paltry as they were, for these services were generally promptly paid; and the doctor could calculate with great certainty on the amount of his income from this business annually, and thus be enabled to adapt his expenditures.—In a country town of 800, or 1000 inhabitants, the births would average perhaps 25, annually. Forty years ago, the fee for attendance in parturition (if promptly paid) was one dollar and a half; if on credit, it was two dollars. With the times the fees have risen from two to five dollars.

In a sparse and scattered population, the physician could not, without great inconvenience, leave the patient after being called to attend a case of parturition, until it terminated. This, and other considerations, prompted him to expedite the business, if possible. He knew that labor would follow the discharge of the waters; so, without regarding the condition of the parts, as soon as it could be effected, he ruptured the membranes, "broke the waters," and then encouraged the patient to exert herself on the recurrence of her pains—to "bear down and help herself." Before this, however, the patient was "seated," or "placed" in such posture or position as was thought to be most appropriate to conduct the labor; there to remain for hours, and it has been even for days, until it terminated; and no entreaty on the part of the patient for a change of posture availed, inasmuch as that in moving, as she was told, "all would go back again."

Shall I be charged with an exaggerated representation of labor forty years ago? "Mark how plain a tale, &c."—for, without going back forty years, while I am writing this letter, the wife of Mr. H. a worthy farmer of one of the oldest towns in my neighborhood, has sought my advice, and given me the following story: Mrs. H., though rather under-size, was a healthy, well-formed woman, 26 years of age. At full period she was taken in labor with her first, and only child, in 1844. The labor commenced on Sabbath morning; her medical attendant, a graduate of a public institution, was immediately called; and, with the exception of about two hours of the forenoon of that day, he remained constantly with the patient until its termination on the following Wednesday, at 10 o'clock A. M. Sometime in the afternoon of the Sabbath, Mrs. H. was "placed" on her side, on the edge of the bed, and her physician assured her that "all was right," and that she might confidently hope to "get to bed" in a short time. The night passed, and the succeeding (Monday) day and night, without relief. On Tuesday the Doctor bethought himself to apply the forceps, but did not succeed, "because the head of the child rolled so." On Wednesday morning, at 1 o'clock, another physician was called in consultation, who coincided in opinion with his friend in attendance, "that all was going on well, and nothing but time was wanting to bring the labor to a favorable issue." Both gentlemen now remained in attendance. But snatching a moment of comparative quiet to refresh themselves with breakfast, in their absence the child was expelled by a natural effort, dead. I should have remarked, that ergot was given freely at different times during the labor. Thus, for nearly seventy hours, with the exception that twice the patient was taken from the bed to be steamed "over a pot of herbs," she remained on her side, exerting herself to the very utmost with every pain. In all this time, neither the bladder nor the bowels were evacuated, nor had she the refreshment of a moment's sleep. The only explanation offered for this cruel delay was, that the mother was *small*, and the child *large*. As might be expected, inflammation, suppuration and sloughing ensued; and after months of indescribable suffering, Mrs. H. is left with a fistulous opening of the urethra into the vagina, and a stricture of the rectum, so that alvine evacuations can be procured only by artificial means.

But gross neglect, and unpardonable delays were not the only evils of country practice, forty years ago—nor are they now. We had then, as we have now, more to dread and deprecate from "hot haste"—from rude, untimely, unfeeling interference. The female who resided at the greatest distance from the accoucheur was comparatively secure from personal injury, for, in a natural labor, if the child was not expelled before the arrival of the Doctor, dilatation would generally have so far advanced as to secure her from the effects of injudicious interposition. But woe betide the woman, especially the youthful, if the Doctor arrived in the first stage of labor! You would be often shocked in the endeavor to recognize the healthy, blooming country girl, in the pale, wan and sickly mother of an only child, if she should have been so fortunate as to secure that alive.

In 1843 I was called to a poor Irish woman, the mother of several children, who had been in labor two days with an arm presentation. Her pains were frequent and forcible. After making all prudent efforts, as I thought, to effect the delivery, without success, it was decided to leave the patient in the care of one attendant until morning,—it being late in the evening—when we would be prepared to adopt such measures as the case might then seem to require. Early on the following morning, I was shocked to find the patient in articulo mortis, and she in fact survived but a few moments after my arrival. The physician who had been in attendance through the night, on particular inquiry, confessed to me that yielding to the intreaties of the poor woman for relief, he resolved to effeet the delivery at all hazard. Accordingly he wrenched off the presenting arm, and forcibly introduced his hand to search for a foot. In the act, the child suddenly escaped from his grasp, and the woman fainted.—She languished about two hours and died. Having permission, we opened the abdomen, and found the child, (minus an arm,) and the placenta, both in the left side among the intestines, having escaped through a rent in the uterus extending from its fundus to the cervix. The rent was on the left side of the uterus, and the operator used his right hand in his attempt to effect the delivery.

The question may be asked—and it is pertinent—what benefit can result from the published recital of these cases—cui bono? I have carefully avoided all personal allusions in the selection of these cases, from an unwillingness to give pain or offence, and the object is, that the history of these and similar instances may prove timely admonitions to young and inexperienced accoucheurs.

*October*, 1845.

